# Utilidad del PSA en vesículas extracelulares como biomarcador de seguimiento en el cáncer de próstata

**DOI:** 10.1515/almed-2025-0159

**Published:** 2025-11-04

**Authors:** Amaia Sandúa, José L. Pérez-Gracia, Estibaliz Alegre, Álvaro González

**Affiliations:** Servicio de Bioquímica, Clínica Universidad de Navarra, Pamplona, España; Laboratorio de Biomarcadores Circulantes en Cáncer, Cáncer Center de la Clínica Universidad de Navarra (CCUN), Pamplona, España; Departamento de Oncología, Clínica Universidad De Navarra, Pamplona, España; Servicio de Bioquímica, Clínica Universidad de Navarra, IdiSNA, Instituto de Investigación Sanitaria de Navarra, Pamplona, España; IdiSNA, Instituto de Investigación Sanitaria de Navarra, Pamplona, España

**Keywords:** biomarcador, respuesta clínica, vesículas extracelulares, antígeno prostático específico, progresión tumoral

## Abstract

**Objetivos:**

El antígeno prostático específico (PSA) circula unido a las vesículas extracelulares (VEs). Los niveles de este tipo de PSA (ev-PSA) se encuentran más elevados en el cáncer de próstata (CaP) que en las patologías benignas y los controles sanos, por lo que el ratio de PSA en vesículas extracelulares/suero (ev/srm) podría servir como biomarcador diagnóstico en el CaP. Evaluamos la utilidad del ev-PSA en el CaP como biomarcador de seguimiento para la detección de recidiva o la evaluación de la respuesta a los tratamientos sistémicos.

**Métodos:**

Se obtuvieron muestras secuenciales (basal, respuesta y progresión) de diez pacientes con CaP avanzado tratados con terapia hormonal o quimioterapia. Las VEs se aislaron del suero mediante cromatografía de exclusión molecular. El PSA total (T-PSA) y el PSA libre (F-PSA) se cuantificaron en suero y VEs en un módulo c602 de un Cobas 8000 (Roche Diagnostics) empleando los inmunoensayos Elecsys, para posteriormente calcular el ratio ev/srm de PSA.

**Resultados:**

Se realizó la cuantificación del T-PSA en VEs (ev-T-PSA) en todas las muestras, arrojando una mediana del ratio ev/srm T-PSA del 1,4 % (Q1-Q3: 1,1–1,9 %). En la respuesta clínica, no se observó un descenso significativo del ev-T-PSA (p=0,055), ni un incremento de los valores del ratio ev/srm T-PSA (p=0,078). Durante la progresión de la enfermedad, el ratio ev/srm T-PSA disminuyó significativamente con respecto a los valores basales (p=0,037) y de respuesta (p=0,008), aunque no se produjeron variaciones en las concentraciones de srm-T-PSA y ev-T-PSA (p=0,625 y p=0,482, respectivamente). El mayor descenso en las concentraciones de srm-T-PSA y ev-T-PSA se observó en pacientes que recibieron terapia hormonal.

**Conclusiones:**

El ratio ev/srm T-PSA podría ser de utilidad en la detección de la progresión tumoral y recidiva en el CaP avanzado. Sin embargo, este ratio presentaría una utilidad limitada como biomarcador de seguimiento en la evaluación de la respuesta clínica al tratamiento hormonal y a la quimioterapia.

## Introducción

El cáncer de próstata (CaP) es el segundo tipo de cáncer más frecuente y la quinta causa de mortalidad relacionada con el cáncer en hombres en todo el mundo [1], 2]. El CaP es una enfermedad clínicamente muy heterogénea y, aunque la mayoría de los pacientes desarrollan un tumor de crecimiento lento que queda confinado a la próstata y/o sobreviven a largo plazo debido a la efectividad de algún tratamiento, existe un subgrupo de pacientes que presentan un fenotipo más agresivo, caracterizado por la progresión metastásica, con resultados clínicos desfavorables [3].

El biomarcador de referencia en el CaP es el antígeno prostático específico (PSA). El PSA es una serino-proteasa sintetizada por las células epiteliales de la glándula prostática [4] cuya cuantificación en sangre se emplea para la detección y el diagnóstico precoz del CaP, dada la relación existente entre los niveles de PSA y un mayor riesgo de presentar la enfermedad. No obstante, a pesar de su especificidad para el tejido prostático, el PSA no presenta especificidad para el cáncer, dado que sus concentraciones también pueden verse incrementadas en patologías benignas como la hiperplasia benigna de próstata (HBP) o la prostatitis, lo cual merma su utilidad como marcador tumoral en el CaP [5], 6]. Así mismo, existen múltiples factores, como la edad, el volumen de la próstata, algunos procedimientos urológicos y ciertos fármacos, entre otros, que pueden influir en las concentraciones de PSA [7], [8], [9]. Se han investigado diversas estrategias destinadas a mejorar la especificidad y precisión diagnóstica del PSA, desde el desarrollo de parámetros relacionados con el PSA (e.g., velocidad del PSA, índice de PSA libre) hasta la identificación de nuevos biomarcadores y el uso de modelos multivariables (e.g., PHI, prueba 4Kscore, PCA3) [10], [11], [12]. Sin embargo, aunque algunos de estos biomarcadores han mostrado un potencial prometedor, su utilidad clínica sigue siendo inconsistente, por lo que es necesario seguir investigando para perfeccionar las estrategias diagnósticas e identificar alternativas de mayor fiabilidad.

El PSA es un biomarcador de elevada sensibilidad para el seguimiento del CaP en pacientes sometidos a terapia radical [13], 14]. Tras una prostatectomía radical exitosa, los niveles de PSA deben ser indetectables, siendo la detección de cualquier concentración indicativa de un mal pronóstico [15]. La posterior elevación del PSA tras lograr concentraciones indetectables indica recidiva bioquímica [16], y un valor de PSA post-cirugía superior a 0,4 μg/L es predictivo de un mayor riesgo de desarrollar nuevas metástasis [17]. La monitorización del PSA también se realiza para evaluar la respuesta a los tratamientos sistémicos en el CaP, entre los que se encuentran la terapia hormonal, la quimioterapia y la inmunoterapia [18]. Sin embargo, aún no se han estandarizado las definiciones de respuesta y progresión de la enfermedad. En los pacientes con CaP metastásico resistente a la castración sometidos a quimioterapia o inmunoterapia, las variaciones en los niveles de PSA presentan un valor predictivo limitado para la supervivencia global [19], 20].

Las vesículas extracelulares (VEs) son pequeñas vesículas lipídicas de membrana secretadas por casi todas las células al espacio extracelular, que desempeñan un papel crucial en la comunicación intercelular en múltiples procesos fisiológicos y patológicos [21], 22]. Las VEs actúan como portadores de biomoléculas específicas de las células progenitoras, como proteínas, ARNmi, ARNm, ARNlnc, y lípidos, que pueden modular las vías de señalización en las células receptoras [23]. La secreción activa de VEs parece estar aumentada en las células tumorales, donde contribuyen a múltiples procesos relacionados con el proceso tumoral [24]. Se pueden encontrar VEs en una amplia variedad de fluidos biológicos, como la sangre y la orina, que se pueden extraer mediante procesos mínimamente invasivos [25], lo que los convierte en potenciales biomarcadores para el diagnóstico, pronóstico y seguimiento de distintas patologías, incluido el CaP [26]. Diversos estudios han demostrado la expresión de PSA en VEs procedentes de la próstata [27], 28] lo que evidencia su posible utilidad como biomarcador diagnóstico [29]. Logozzi y col. [30] observaron niveles significativamente más elevados de VEs CD81+ PSA+ en pacientes con CaP, frente a los individuos con HBP y sujetos sanos, lo que sugiere su utilidad en el cribado del CaP y el diagnóstico precoz. Así mismo, en investigaciones anteriores, nuestro equipo identificó mayores concentraciones de PSA unido a VEs (ev-PSA) en pacientes con CaP, frente a los sujetos sanos o con HBP [31]. Además, demostramos un mejor rendimiento diagnóstico del ev-PSA, concretamente, del ratio ev/srm de PSA, con respecto al índice de PSA libre sérico, que es empleado habitualmente en la práctica clínica rutinaria. Aparte del diagnóstico, las VEs y su carga molecular también se podrían emplear en el CaP para la estadificación, el pronóstico, y el seguimiento de la progresión de la enfermedad y la respuesta a la terapia [26]. Además, en el CaP avanzado, se han investigado biomarcadores de biopsia líquida, como el AR-V7 cuantificado en células tumorales circulantes (CTC) [32], 33] y el ADN tumoral circulante en pacientes resistentes a la castración [34], para predecir la resistencia a la terapia hormonal.

En el presente estudio, investigamos el impacto de las diferentes terapias para el CaP avanzado en la liberación de PSA en las VEs, y evaluamos el potencial del ev-PSA como biomarcador de seguimiento para la detección de recidivas o la monitorización de la respuesta a los tratamientos sistémicos. Para tal fin, analizamos muestras recogidas secuencialmente de pacientes con CaP avanzado sometidos a diferentes terapias.

## Materiales y métodos

### Muestras y selección de pacientes

Se seleccionaron diez pacientes con adenocarcinoma de próstata avanzado del Departamento de Oncología Médica, que habían recibido terapia hormonal o quimioterapia (Tabla 1). De acuerdo al protocolo aprobado por el Comité de Ética de la Universidad de Navarra (código 2010.111), se recogieron muestras secuenciales tras obtener el consentimiento informado en diferentes momentos de la evolución de la enfermedad: al inicio del estudio y en el momento de la progresión tumoral para todos los participantes, y además en el momento de la respuesta clínica al tratamiento antes de la progresión de la enfermedad en ocho de estos pacientes. El tiempo medio transcurrido entre la extracción de las muestras basales y las de respuesta clínica fue de 4 meses, mientras que las muestras de la progresión se obtuvieron 13 meses después de iniciar el tratamiento. La única excepción fue el paciente 2, cuya muestra de progresión se obtuvo 50 meses después del inicio del tratamiento. El estudio se llevó a cabo de acuerdo a los principios éticos para la investigación médica descritos en la Declaración de Helsinki.

**Tabla 1:** Características clínicas de los participantes del estudio. Los datos de edad se presentan como mediana y rango intercuartílico.

n	10
Edad, años	68 (65–71)
Gleason	
≤7	3
>7	5
No disponible	2
ISUP	
<3	2
≥3	5
No disponible	3
Estadio	
III	2
IV	8
Tratamiento	
Terapia hormonal	7
Quimioterapia	3

Las muestras de sangre se recogieron en tubos de recogida de suero BD Vacutainer de 5 mL (Beckton Dickinson, East Rutherford, USA). Para obtener el suero, los tubos se centrifugaron a 2000×*g* durante 10 minutos tras la formación del coágulo. A continuación, las muestras séricas se alicuotaron y se almacenaron a −80 °C hasta su posterior análisis.

La determinación del tipo histológico, la puntuación de Gleason e ISUP, el estadio clínico y los tratamientos se obtuvieron de las historias médicas de los pacientes y se basaron en pruebas clínicas, analíticas y de imagen de acuerdo con las guías clínicas vigentes.

### Aislamiento de vesículas extracelulares

Las VEs fueron aisladas del suero mediante cromatografía de exclusión molecular empleando el kit comercial de mini-columnas Exo-spin^TM^ (Cell Guidance System, Cambridge, Reino Unido). Tras la descongelación, las muestras séricas se centrifugaron a 16,000×*g* durante 30 minutos.

Se recogieron 100 µL de sobrenadante con VEs y se aplicaron a la columna Exo-spin previamente estabilizada y preparada con tampón fosfato salino (PBS). Por último, la fracción de VEs se eluyó de la columna con 180 µL de PBS y se diluyó hasta alcanzar un volumen final de 200 µL. Para el cálculo de la concentración final de PSA, se tuvo en cuenta el factor de corrección de la dilución.

Este método de aislamiento había sido validado previamente para la extracción de VEs séricas, y la caracterización de las VEs aisladas está descrita en una publicación anterior [31].

### Cuantificación del PSA

Las concentraciones de PSA total (T-PSA) y PSA libre (F-PSA) tanto en suero (srm-) como en las VEs aisladas (ev-) se determinaron en un módulo c602 de un Cobas 8000 (Roche Diagnostics, Basel, Suiza) empleando los inmunoensayos de electroquimioluminiscencia Elecsys^®^ total PSA y Elecsys^®^ free PSA, diseñados para la cuantificación sérica. Los límites de detección fueron de 0,010 μg/L para el T-PSA y 0,016 μg/L para el F-PSA, con límites de cuantifación de 0,014 μg/L y 0,018 μg/L, respectivamente.

Para calcular los ratios ev/srm (%) de T-PSA y F-PSA, se aplicaron las siguientes fórmulas:

$$\text{Ratio}\;\text{evsrm }\text{T-PSA }\left(\%\right)=\text{ev-T-PSA}/\text{srm-T-PSA}\times 100.$$

$$\text{Ratio ev}\text{srm}\;\text{F-PSA }\left(\%\right)=\text{ev-F-}\text{PSA}/\text{srm-F-PSA}\times 100.$$

### Análisis estadístico

El análisis estadístico se realizó con el programa Graphpad Prism versión 6 aplicando métodos no paramétricos. Los datos se representaron como mediana y rango intercuartílico. Para realizar el análisis comparativo, se emplearon las pruebas de Mann-Whitney U test y la prueba de Wilcoxon, mientras que las correlaciones se determinaron con la prueba de Spearman. Se consideró significación estadística un valor de p bilateral <0,05.

## Resultados

### Cuantificación del PSA en vesículas extracelulares

La mediana de la concentración del srm-PSA fue de 14,8 μg/L para T-PSA (Q1-Q3: 6,0–109,3 μg/L) y 4,0 μg/L para F-PSA (Q1-Q3: 0,7–23,7 μg/L). Se cuantificó el ev-T-PSA en todas las muestras con una mediana de 0,177 μg/L (Q1-Q3: 0,102–1,006 μg/L). Por otro lado, el ev-F-PSA se detectó en el 96 % de las muestras analizadas con una mediana de 0,074 μg/L (Q1-Q3: 0,034–0,224 μg/L). Se obtuvieron valores similares del ratio ev/srm para el T-PSA (mediana 1,4 %; Q1-Q3: 1,1–1,9 %) y F-PSA (mediana 1,9 %; Q1-Q3: 0,8–4,6 %).

Se observó una correlación significativa entre las concentraciones de ev-PSA tanto para el T-PSA (r=0,958; p<0,001) como para el F-PSA (r=0,879; p<0,001).

### Análisis del PSA en vesículas extracelulares en muestras secuenciales

**Figura 1:**
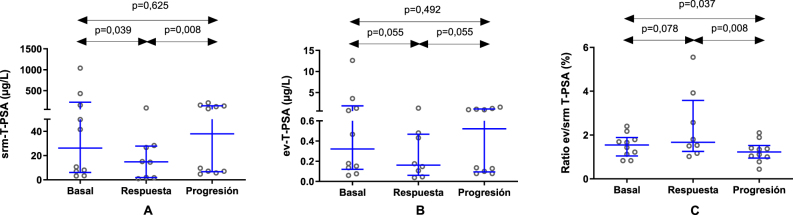
Análisis comparativo de las concentraciones de T-PSA en suero (srm) (A) y en vesículas extracelulares (ev) (B), así como del ratio ev/srm T-PSA (C) en condiciones basales, durante la respuesta al tratamiento y en la progresión de la enfermedad en 10 pacientes con cáncer de próstata avanzado. T-PSA, PSA total.

En primer lugar, al analizar el T-PSA en la respuesta clínica, las concentraciones séricas disminuyeron significativamente con respecto al valor basal, de una mediana inicial de 26,1 μg/L (Q1-Q3: 6,1–222,7 μg/L) a 14,8 μg/L (Q1-Q3: 1,8–27,9 μg/L; p=0,039) (Figura 1A). Sin embargo, la disminución observada en las concentraciones de ev-T-PSA no alcanzó la significación estadística (p=0,055) (Figura 1B) ni tampoco el incremento del ratio ev/srm T-PSA (p=0,078) (Figura 1C).

Tras mostrar respuesta a los diferentes tratamientos y la consiguiente mejora de los resultados clínicos, la totalidad de los pacientes experimentaron una recidiva. Los niveles de srm-T-PSA se incrementaron significativamente durante la progresión del tumor (mediana: 38,0 μg/L; Q1-Q3: 6,7–137,9 μg/L; p=0,008) (Figura 1A), y aunque el ev-T-PSA no varió (p=0,055) (Figura 1B), el ratio ev/srm T-PSA disminuyó significativamente (mediana: 1,2 %; Q1-Q3: 0,9–1,5 %), en comparación con los niveles observados durante la respuesta clínica (mediana: 1,7 %; Q1-Q3: 1,3–3,6 %; p=0,008) (Figura 1C).

Si comparamos los resultados en el momento de la progresión de la enfermedad con los valores basales, podemos observar una disminución significativa en los valores del ratio ev/srm T-PSA (p=0,037).

Sin embargo, no observamos cambios significativos en las concentraciones de srm-T-PSA y ev-T-PSA (p=0,625 y p=0,482, respectivamente).

**Figura 2:**
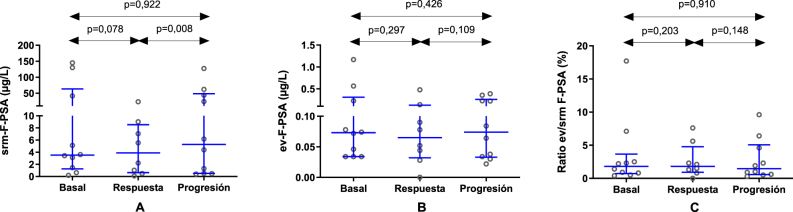
Análisis comparativo de las concentraciones de F-PSA en suero (srm) (A) y en vesículas extracelulares (ev) (B), así como del ratio ev/srm F-PSA (C) en condiciones basales, durante la respuesta al tratamiento y en la progresión de la enfermedad en 10 pacientes con cáncer de próstata avanzado. F-PSA, PSA libre.

En las muestras de los mismos pacientes, las concentraciones de srm-F-PSA mostraron una tendencia similar a la del srm-T-PSA durante la evolución de la enfermedad, aunque únicamente las variaciones entre la respuesta a la terapia y la progresión alcanzaron significación estadística, ya que los niveles aumentaron de 3,9 μg/L (Q1-Q3: 0,6–8,5 μg/L) a 5,3 μg/L (Q1-Q3: 0,5–48,2 μg/L; p=0,008). En relación con el ev-F-PSA (Figura 2B), tal como ocurrió con el ev-T-PSA, los cambios no fueron significativos ni durante la respuesta clínica (p=0,297) ni durante la progresión (p=0,109). Aunque los valores del ratio ev/srm F-PSA se incrementaron durante la respuesta para posteriormente disminuir durante la progresión, tal como se esperaba, las diferencias no fueron significativas en ningún momento de la evolución clínica de la enfermedad (p=0,203 y p=0,148, respectivamente) (Figura 2C). A este respecto, no se observaron variaciones significativas al comparar los valores durante la progresión con los valores basales de srm-F-PSA, ev-F-PSA, y del ratio ev/srm F-PSA (p=0,922, p=0,426 y p=0,910, respectivamente).

### Análisis del PSA sérico y en vesículas extracelulares según el tratamiento recibido

**Figura 3:**
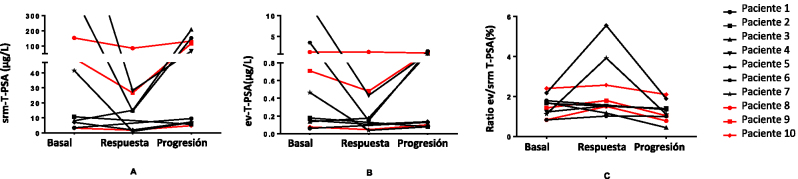
Representación de los cambios absolutos en las concentraciones de T-PSA en suero (srm) (A) y en vesículas extracelulares (ev) (B), así como en los valores del ratio ev/srm T-PSA (C) durante la respuesta al tratamiento y en la progresión de la enfermedad en 10 pacientes con cáncer de próstata avanzado. T-PSA, PSA total.

Analizamos los resultados de cada paciente con el objeto de investigar una posible relación entre las variaciones en el ev-PSA y el tipo de tratamiento recibido (Figuras 3 y 4). Los pacientes 1 a 7 recibieron terapia hormonal, mientras que los pacientes 8 a 10 fueron tratados con quimioterapia.

**Figura 4:**
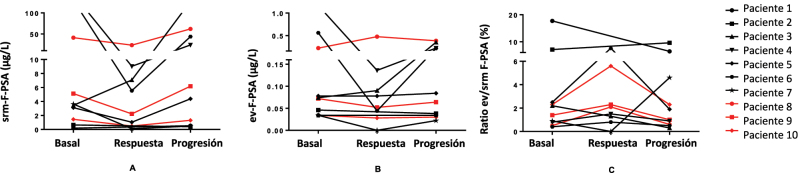
Representación de los cambios absolutos en las concentraciones de F-PSA en suero (srm) (A) y en vesículas extracelulares (ev) (B), así como en los valores del ratio ev/srm F-PSA (C) durante la respuesta al tratamiento y en la progresión de la enfermedad en 10 pacientes con cáncer de próstata avanzado. F-PSA, PSA libre.

Observamos que todos los pacientes seguían la misma tendencia durante la respuesta al tratamiento: una disminución en las concentraciones de srm-T-PSA y ev-T-PSA, sumado a un incremento del ratio ev/srm T-PSA, a excepción del paciente 3, que mostró el comportamiento contrario (Figura 3). Tal como se esperaba, durante la progresión de la enfermedad, las concentraciones de srm-T-PSA y ev-T-PSA aumentaron, mientras que el ratio ev/srm T-PSA disminuyó con respecto a la respuesta en todos los pacientes excepto en el paciente 8, cuya concentración de ev-T-PSA también disminuyó durante la progresión. Además, los cambios en el ev-T-PSA durante la progresión fueron por lo general menos marcados en aquellos que presentaban menores concentraciones de srm-T-PSA.

Si comparamos las concentraciones en la progresión con respecto a los valores basales, el 40 % de los pacientes mostraron un aumento de los niveles de srm-T-PSA y ev-T-PSA, mientras que 50 % de los pacientes presentaron una disminución, y un paciente (paciente 5) mostró un leve incremento de las concentraciones de srm-T-PSA y una disminución de ev-T-PSA. El ratio ev/srm T-PSA disminuyó en el 80 % de los participantes, siendo los pacientes 4 y 6 la excepción.

De la totalidad de los pacientes, las mejores respuestas bioquímicas, evidenciadas por la disminución tanto de los niveles de srm-T-PSA como de ev-T-PSA, se observaron en tres pacientes que recibieron terapia hormonal: el paciente 4 (97,3 y 96,6 %, respectivamente); el paciente 6 (96,6 y 95,8 % respectivamente) y el paciente 7 (97,7 y 91,8 % respectivamente). El mayor aumento en el ratio ev/srm T-PSA se observó en el paciente 5 (156 %) y el paciente 7 (250 %). Durante la progresión, la variación más significativa en las concentraciones de srm-T-PSA con respecto a la respuesta se observó también en los pacientes sometidos a terapia hormonal: el paciente 3 (1,282 %), el paciente 6 (947 %) y el paciente 7 (625 %). Cabe señalar que el paciente 6 también fue el que experimentó el mayor aumento de ev-T-PSA, por lo que su índice se mantuvo constante durante la progresión. Por otro lado, en aquellos pacientes tratados con quimioterapia (paciente 8, paciente 9 y paciente 10), observamos una menor variación en las concentraciones de PSA en suero y en VEs, aunque siguieron la tendencia esperada durante la evolución de la enfermedad. La excepción fue el paciente 8, cuyas concentraciones de ev-T-PSA no disminuyeron durante la respuesta, permaneciendo constantes.

Las concentraciones de srm-F-PSA y de ev-F-PSA, así como los valores del ratio ev/srm F-PSA mostraron una tendencia similar a la del T-PSA durante la evolución de la enfermedad en casi todos los pacientes (Figura 4). Las excepciones fueron los pacientes 7, cuyo ratio ev/srm F-PSA, a diferencia del T-PSA, disminuyó durante la respuesta, para posteriormente aumentar sustancialmente, y el paciente 2, en el que se observaron resultados discrepantes entre el ratio ev/srm T-PSA y el ratio ev/srm F-PSA.

## Discusión

En el CaP, realizar un seguimiento estrecho de la respuesta tumoral al tratamiento, así como la detección precoz de recidivas resultan de vital importancia, dado que no es poco frecuente que se produzca una recaída de la enfermedad incluso años después de su curación. El PSA ha sido empleado como biomarcador de progresión [10], siendo considerado un marcador fiable de recurrencia y/o aparición de nuevas metástasis [13], 14]. Además, en el CaP avanzado, donde los tratamientos como la terapia hormonal, la quimioterapia o la inmunoterapia podrían no erradicar la enfermedad completamente, la monitorización del PSA se ha utilizado para evaluar la respuesta tumoral a lo largo del tiempo, determinar la eficacia del tratamiento y decidir cuándo cambiar de terapia [18] (https://www.cancer.org/, visitado el 21 de junio de 2023). Sin embargo, tal como se ha indicado en otras publicaciones [19], 20], el empleo de PSA sérico presenta ciertas limitaciones destacables, por lo que es preciso identificar otros biomarcadores que proporcionen información específica sobre las variaciones en el tejido tumoral en respuesta a las diferentes terapias sistémicas.

En el presente estudio, el análisis del srm-T-PSA en muestras sucesivas arrojó los resultados esperados, esto es, una relación entre la mejoría clínica y la respuesta al tratamiento y una disminución significativa de los niveles de marcadores tumorales séricos, que posteriormente aumentaron cuando se produjo progresión de la enfermedad. Aunque las concentraciones de ev-T-PSA mostraron una tendencia similar, los cambios no alcanzaron la significación estadística en ningún momento durante la evolución de la enfermedad. Durante la progresión del tumor, los valores del ratio ev/srm T-PSA se redujeron significativamente, en comparación con los observados durante la respuesta clínica y al inicio del tratamiento. La disminución de los valores del ratio ev/srm T-PSA durante la progresión respaldaría la hipótesis de que la incorporación del PSA a las VEs y su posterior liberación desde la próstata a la circulación es más complejo que la del PSA soluble. Este proceso depende de la producción y la integridad estructural del tejido, que podrían ser modulados por el microambiente tumoral y la actividad celular [28]. Durante la progresión de la enfermedad, la mayor parte del PSA producido por el tejido tumoral prostático se liberaría a la circulación en su forma soluble, lo que explicaría el incremento significativo en las concentraciones de srm-T-PSA, pero no de ev-T-PSA. Como resultado, se produjo una disminución del ratio ev/srm T-PSA. Además, dichas diferencias en la liberación de ambas formas moleculares del PSA se intensificaron a media que las concentraciones de srm-T-PSA aumentaban. En general, en aquellas muestras con niveles más bajos de srm-T-PSA, la proporción de PSA liberado en las VEs fue más parecida a la del PSA soluble, con valores del ratio ev/srm T-PSA más altos. Estos hallazgos coinciden con observaciones anteriores publicadas por nuestro grupo [31], en las que se indica que el ratio ev/srm T-PSA fue notablemente superior en los pacientes con srm-T-PSA <4 μg/L, que aquellos que superaban este punto de corte. Esto sugiere que el ratio ev/srm T-PSA podría ser una herramienta útil para la detección precoz de progresión tumoral, tal como muestran los resultados del paciente 2. En dicho paciente, el ratio ev/srm T-PSA disminuyó durante la progresión, mientras que las concentraciones de PSA en suero y VEs no aumentaron como se esperaba, lo que implica que dichas concentraciones podrían no aportar información útil sobre la situación clínica del paciente. Cabe mencionar que en este paciente fue donde el intervalo de tiempo transcurrido entre la muestra basal y la progresión fue más prolongado, sugiriendo que este nuevo biomarcador podría ser de especial utilidad en estos casos.

Por otro lado, a pesar de que las concentraciones de srm-T-PSA disminuyeron durante la respuesta clínica a la terapia, no se observó una disminución significativa del ev-T-PSA, ni un aumento del ratio ev/srm T-PSA. Estos hallazgos sugieren que el PSA unido a VEs, una vez liberado, permanece en la circulación durante más tiempo que el PSA soluble, lo que implicaría una vida media plasmática más prolongada. Consecuentemente, la disminución de las concentraciones de ev-PSA no sería tan significativa como la del PSA soluble en el mismo momento de análisis. Esto queda evidenciado en los resultados del paciente 8, ya que a pesar de haber experimentado una disminución del srm-T-PSA durante la respuesta clínica, el ev-T-PSA permaneció constante, derivando en un incremento del ratio ev/srm T-PSA. Sería interesante profundizar en esta hipótesis analizando la cinética de eliminación del PSA unido a VEs con el fin de caracterizar mejor esta nueva forma de PSA y determinar su función distintiva frente al PSA soluble en la progresión tumoral y el seguimiento clínico. Con respecto a los resultados de F-PSA, no se produjeron variaciones significativas, lo que indica que ni las concentraciones de ev-F-PSA ni el ratio ev/srm F-PSA serían biomarcadores de CaP adecuados durante la evolución de la enfermedad.

Al analizar los resultados de los pacientes según el tipo de terapia recibida, observamos que los pacientes que recibieron terapia hormonal experimentaron mayores variaciones en las concentraciones de PSA sérico, tanto en el momento de la respuesta como durante la progresión, frente a aquellos tratados con quimioterapia. Sin embargo, no observamos ninguna relación significativa entre el tipo de tratamiento y las variaciones analíticas durante la progresión en los resultados de ev-T-PSA, aunque los pacientes tratados con terapia hormonal parecieron experimentar una mayor disminución de las concentraciones de ev-T-PSA en la respuesta. En cuanto al ratio ev/srm T-PSA, no identificamos ninguna relación entre el tratamiento recibido y los cambios en los valores del ratio en ningún momento de la evolución de la enfermedad.

Nuestros resultados sirven como prueba de concepto, realizada en una pequeña cohorte de pacientes con CaP avanzado y con regímenes de tratamiento heterogéneos, lo que constituye la principal limitación del estudio. Para conocer en profundidad la posible utilidad del ev-PSA en la monitorización de la respuesta a diferentes tipos de terapias y la detección de la progresión tumoral, el primer paso esencial sería reclutar una cohorte más amplia de pacientes, lo que mejoraría tanto la potencia estadística como la capacidad de generalización de los resultados. Así mismo, incluir poblaciones homogéneas de pacientes según el tipo de terapia recibida resulta esencial, ya que se minimizarían las variables de confusión y se reducirían los sesgos, permitiendo obtener conclusiones más precisas y robustas. Además, extender el estudio para incluir otras modalidades de tratamiento como la inmunoterapia, un campo cada vez más importante y objeto de numerosos ensayos clínicos actuales en oncología, podría aportar un valor añadido significativo. De este modo, el diseño de estudios prospectivos que cumplieran los criterios descritos contribuiría a validar nuestros hallazgos, reforzando su relevancia clínica. Por otro lado, el uso de protocolos que garantizaran una obtención, procesamiento y conservación de muestras estandarizadas ayudaría a minimizar la variabilidad entre las muestras de los pacientes, garantizando una mayor consistencia de los datos permitiendo en consecuencia obtener unas conclusiones más sólidas con respecto a la utilidad clínica del ev-PSA en el seguimiento del CaP. Finalmente, la integración biomarcadores adicionales, como otras proteínas asociadas a las VEs o marcadores de ARN, así como la identificación de correlaciones entre la carga de las VEs procedentes de la próstata y técnicas de imagen como el PET-PSMA, contribuirían a una comprensión más completa de la progresión tumoral y serían de especial interés para futuras investigaciones. En conclusión, los resultados obtenidos indican que el ratio ev/srm T-PSA puede servir como un valioso indicador de progresión tumoral, y podría ser útil para detectar recaídas en pacientes con CaP avanzado. No obstante, ni las concentraciones de ev-T-PSA ni el ratio ev/srm T-PSA serían adecuados como biomarcadores de seguimiento de la respuesta clínica a los tratamientos hormonales o la quimioterapia.
